# Developing Evidence to Decision Frameworks and an Interactive Evidence to Decision Tool for Making and Using Decisions and Recommendations in Health Care

**DOI:** 10.1002/gch2.201700081

**Published:** 2018-01-10

**Authors:** Sarah E. Rosenbaum, Jenny Moberg, Claire Glenton, Holger J. Schünemann, Simon Lewin, Elie Akl, Reem A. Mustafa, Angela Morelli, Joshua P. Vogel, Pablo Alonso‐Coello, Gabriel Rada, Juan Vásquez, Elena Parmelli, A. Metin Gülmezoglu, Signe A. Flottorp, Andrew D. Oxman

**Affiliations:** ^1^ Centre for Informed Health Choices Norwegian Institute of Public Health Postboks 4404 Nydalen ,N‐0403 Oslo Norway; ^2^ Global Health Unit Norwegian Institute of Public Health PO Box 4404, Nydalen ,N‐0403 Oslo Norway; ^3^ Department of Health Research Methods Evidence, and Impact (formerly “Clinical Epidemiology and Biostatistics”) McMaster University 1280 Main Street W Hamilton ON L8S 4K1 Canada; ^4^ Norwegian Institute of Public Health, and South African Medical Research Council, Health Systems Research Unit PO Box 19070 ,7505 Tygerberg South Africa; ^5^ Department of Internal Medicine American University of Beirut Medical Center P.O. Box: 11‐0236, Riad‐El‐Solh Beirut ,1107 2020 Beirut Lebanon; ^6^ Division of Nephrology and Hypertension Outcomes and Implementation Research University of Kansas Medical Center 3901 Rainbow Blvd, MS3002 Kansas City KS 66160 USA; ^7^ InfoDesignLab ‐ Sentralen Øvre Slottsgate 3 N‐0157 Oslo Norway; ^8^ UNDP/UNFPA/UNICEF/WHO/World Bank Special Programme of Research Development and Research Training in Human Reproduction (HRP) Department of Reproductive Health and Research, World Health Organization 20 Avenue Appia ,CH‐1211 Geneva Switzerland; ^9^ Iberoamerican Cochrane Center IIB Sant Pau‐CIBERESP Sant Antoni Maria Claret 167 ,08025 Barcelona Spain; ^10^ Evidence Centre Pontificia Universidad Católica de Chile Santiago Chile; ^11^ Department of Internal Medicine Faculty of Medicine Pontificia Universidad Católica de Chile Santiago Chile; ^12^ Epistemonikos Foundation Diagonal Paraguay 362 Santiago Chile; ^13^ Epistemonikos Foundation Arrayán 2735, Providencia Santiago 7510069 Chile; ^14^ Department of Epidemiology of the Lazio Region – ASL Roma 1 Via Cristoforo Colombo 112 ,00147 Rome Italy; ^15^ Norwegian Institute of Public Health Institute of Health and Society, University of Oslo Postboks 4404 Nydalen ,N‐0403 Oslo Norway

**Keywords:** communication, decision making, guidelines, human‐centered design, recommendations

## Abstract

Evidence‐informed health care decisions and recommendations need to be made systematically and transparently. Mediating technology can help manage boundaries between groups making decisions and target audiences, enhancing salience, credibility, and legitimacy for all. This article describes the development of the Evidence to Decision (EtD) framework and an interactive tool to create and use frameworks (iEtD) to support communication in decision making. *Methods*: Using a human‐centered design approach, we created prototypes employing a broad range of methods to iteratively develop EtD framework content and iEtD tool functionality. *Results*: We developed tailored EtD frameworks for making evidence‐informed decisions and recommendations about clinical practice interventions, diagnostic and screening tests, coverage, and health system and public health options. The iEtD tool provides functionality for preparing frameworks, using them in group discussions, and publishing output for implementation or adaption. EtD and iEtD are intuitive and useful for producers and users of frameworks, and flexible for use across different types of topics, decisions, and organizations. They bring valued structure to panel discussions and transparency to published output. *Conclusion*: EtD and iEtD can resolve some of the challenges inherent in multicriteria, multistakeholder decision systems. They are freely available online for all to use at https://ietd.epistemonikos.org/ and https://gradepro.org.

## 1 Introduction

This article is part of a collection of articles exploring how knowledge from scientific advisory committees (SACs) might more effectively inform action. The collection focuses on two chief actors and their relationship: the SAC (a group of individuals with some kind of expertise who provide advice to decision makers based on evidence) and the target users of their advice. In this article, we draw attention to a third technological “actor:” mediating technology. The Evidence to Decision (EtD) framework and the interactive Evidence to Decision (iEtD) tool are technologies designed to be used by SACs, advising about treatment or intervention options in the health sector or by other groups, including target audiences, using that advice for decision making. The purpose of these technologies is to support systematic and transparent use of evidence in decision making, as well as mediate and manage information boundaries between multiple stakeholders with diverging perspectives and information needs. Here, we describe the methods we used to develop the framework and the interactive tool, their primary features and functions, what insights during development contributed to their evolution, and what lessons from this work might inform others designing technology to link knowledge to action.

Decisions and recommendations about health and health care for a population are often made by groups of stakeholders, brought together by organizations (e.g., guideline developers) that publish output for targeted audiences (e.g., health professionals). These processes are often complex, requiring judgments about multiple factors, such as whether the balance of benefits and harms favor one of the management options. Using a systematic approach can help ensure that people making judgments have considered all the important factors (criteria) and that these judgments are informed by the best available evidence.^1^

In addition, transparency is necessary to help ensure that people who are affected (e.g., clinicians, public health agents, health system administrators, patients) know which criteria and supporting evidence were considered, what judgments were made, and the applicability of the decision or recommendation to themselves or their context. Transparency can also increase credibility, enabling people to make an assessment of how much confidence they can have in the result.

However, transparency alone is not sufficient. Effective dissemination of health care decisions or guideline recommendations relies on many other factors, some of which relate to how the information is presented. A systematic review of features that could improve guideline dissemination suggests that presentation strategies, such as increasing usability (ease of use), understandability, and communicability, are among those likely to be important;^2^ examples include targeted summaries for different audiences available in both printed and digital formats.

Dealing with uncertainties is a challenge for any SAC. In 2000, the GRADE Working Group began developing a systematic and transparent approach for making recommendations about health care and health system interventions that included strategies for incorporating and communicating uncertainties. The GRADE approach involves systematically assessing four key evidence‐to‐decision criteria: (1) the balance between desirable and undesirable effects, (2) quality (certainty) of the evidence, (3) variability in people's values and preferences, and (4) resource use, to grade recommendations according to their strength (weak/conditional or strong).^3^, ^4^ The strength of a recommendation reflects the extent to which we can be confident that the desirable consequences of an intervention outweigh the undesirable consequences.^5^ The GRADE working group also identified other factors that can sometimes play a role, such as – in the case of priority setting – the prevalence of the health problem or considerations of equity.^4^ Over 100 organizations have adopted the GRADE approach, including the World Health Organization (WHO), the Cochrane Collaboration, and the National Institute for Health and Care Excellence (NICE).

In 2011, the GRADE working group established the DECIDE project, a European Union funded initiative that aimed to develop targeted dissemination strategies for improving communication of evidence‐based guidelines to decision makers, policy makers, health professionals, patients, and the general public.^6^, ^7^ During initial brainstorming sessions, investigators discussed how the four key GRADE criteria might form the basis of a more comprehensive framework that could provide increased structure and transparency to a broad range of decisions and recommendations in the context of clinical care, diagnostic and screening tests, health care coverage, health systems, and public health, while supporting communication to target audiences.

The concept of an EtD grew out of these discussions and earlier work by the GRADE Working Group on evidence to decision criteria.^8^, ^9^, ^10^ Many other sets of criteria for making different types of decisions already existed; although we used these to inform our criteria choices while developing EtD, none of them were based explicitly on the GRADE approach.^11^, ^12^, ^13^, ^14^

Our overarching purpose was to support three different types of decisions: moving from evidence to recommendations (e.g., creating clinical guidelines), moving from evidence to decisions (e.g., making a coverage decision), and moving from recommendations to decisions (e.g., reassessing an international recommendation for a national setting). We also aimed to support communication to people who would use these decisions in some way (for implementation or adaptation) or people whom these decisions affected, such as regional managers, health professionals, and their patients.

Our work was informed by Daniels' framework of accountability for reasonableness, a “fair process” approach to priority setting with three key elements: “transparency,” “buy‐in from key stakeholders,” and “revisability in light of new evidence or judgments.”^15^ We sought to help decision makers achieve fairness in their decision making by creating tools that would facilitate these three process elements. The EtD framework aimed to:

(1) Help guideline panels or other decision makers use evidence in a systematic and transparent way to inform their deliberations when making recommendations and decisions by:

- Ensuring transparent consideration of the most important criteria for making a recommendation or decision;
- Presenting a concise understandable summary of the best available evidence to inform judgments about each criterion;
- Helping structure discussion and identify reasons for disagreements;
- Facilitating later adaptation of recommendations and decisions to specific contexts other than the ones for which they were developed originally.

(2) Support people affected by a decision or recommendation by:

- Enabling them to understand the criteria, evidence, and judgments that led to the conclusions;
- Helping them decide whether recommendations can and should be implemented in their own setting.

In this article, we describe how we developed the EtD framework and the iEtD tool, an online solution for creating and using frameworks.

## 2 Methods

Although the framework and tool name is “Evidence to Decision,” these have a broader context of use including both recommendations and decisions. From now on, we use “decision” to mean both decisions and recommendations. See **Table** **1** for a definition of all article terms.

**Table 1:** Definition of terms

Term	Definition
Decision	Both decisions and recommendations.
Organization	Entity responsible for making decisions or recommendations.
Technical team	People collecting and appraising evidence and preparing frameworks.
Panel	Group making decisions.
Chair	Person managing panel meetings.
User	Anyone using EtD or iEtD, including technical teams, chairs, panels, and end users.
End user	People accessing EtD or iEtD output to read or to reuse in new decisions.
Stakeholder	Anyone who has an interest in the use, input, or output of EtD or iEtD, but who is not directly a user.
EtD, EtD framework	Evidence to Decision framework. We often use the singular tense “framework” to signal that this is one umbrella concept. In fact, EtD is a set of closely related frameworks based on a common set of criteria, each tailored slightly to a different type of question.
iEtD, iEtD tool	Interactive Evidence to Decision framework tool. An online tool that facilitates tailoring, preparation, and use of EtD frameworks.

### 2.1 Overarching Approach

**Figure 1:**
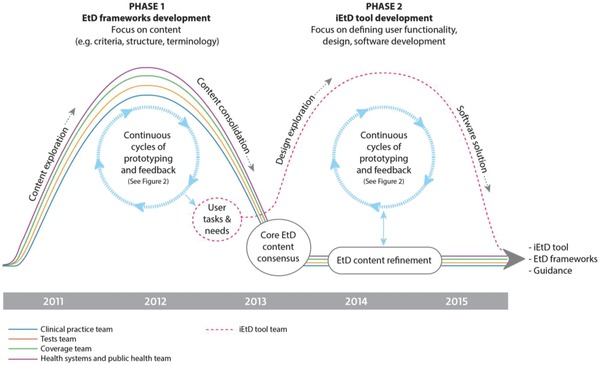
Development timelines for the EtD frameworks and the iEtD tool.

The project evolved in two phases over five years (**Figure** **1**). Four project development teams worked in parallel in four domains (clinical practice, coverage, tests, and health system and public health). They included health researchers from nine DECIDE project partner institutions and seven countries,^7^ with expertise in GRADE methodology, evidence synthesis, guideline production, information design, and software development. A fifth team exploring patients' and public perspectives on guideline output informed our work.

In Phase 1, the teams focused on developing EtD framework content for their respective topic areas. In Phase 2, teams continued to refine content and test frameworks in real‐life guideline panels,^16^ while a smaller group of researchers, designers, and software developers carried out the development of the iEtD tool based on findings (content, user tasks, and needs) from Phase 1.

**Figure 2:**
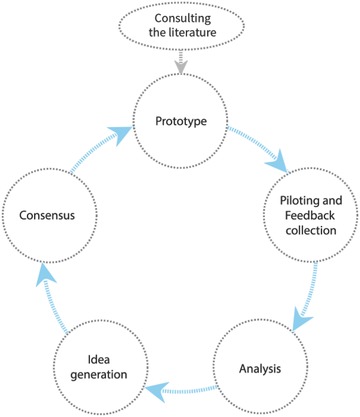
Continuous cycles of prototyping and feedback in both Phases 1 and 2.

The project teams' development efforts were grounded in a human‐centered design approach.^6^, ^17^, ^18^, ^19^ Here, the needs of multiple users and key stakeholders drove incremental improvements in continuous cycles of prototyping, piloting and feedback collection, analysis, idea generation, and consensus (**Figure** **2**). We sought to achieve a balance of resolving user and stakeholder concerns while preserving our original objectives.

Below is a summarized description of our approach. See Additional files S1 and S2 (Supporting Information) for more details about the methods and participants that informed development cycles.

### 2.2 Consulting the Literature and Prototyping

Following a consultation of existing systematic reviews of decision making frameworks,^11^, ^12^, ^13^, ^14^ teams worked in parallel to develop EtD framework prototypes for their respective domains. We populated prototypes with examples and focused on establishing core content (e.g., criteria, document structure and terminology). A criterion was considered important if it had the potential to change direction or strength of the decision or recommendation. We tested assumptions about importance by looking for examples of decisions or recommendations where a proposed criterion had changed conclusions. If we could not find such a case, we did not include that criterion. We collected feedback that informed prototype changes (see more detail below). Teams shared ideas and findings through monthly Skype meetings, where team leaders discussed progress and concerns, and worked to consolidate approaches.

### 2.3 Piloting and Collecting Feedback

We aimed to learn about the needs and concerns of multiple types of users and stakeholders: people in organizations involved in decision making and dissemination (e.g., guideline producers, panel members) as well as people who would use this information (e.g., policy makers, health professionals, the public). Guided largely by themes from a framework of user experience,^17^ we collected both positive and negative feedback, perceived problem areas, barriers, facilitators, and suggestions that could inform incremental improvement of the EtD frameworks and iEtD tool across Phase 1 and Phase 2 (Figure 1). To achieve this, we used a broad range of structured, semistructured, and open ended methods to inform cycles of prototyping and feedback: piloting in actual guideline projects,^20^ participatory and nonparticipatory observation of guideline panels and workshops, prototype sketching, testing examples (entering different kinds of content into prototypes), user‐test interviews, stakeholder feedback, questionnaires, surveys,^21^, ^22^ and discussion in face‐to‐face meetings. Users and stakeholders involved in development work came from many regions, recruited through partner countries in Europe or through WHO, GRADE, Cochrane, or Guidelines International Network.

### 2.4 Analysis, Idea Generation, Consensus, New Prototypes

We analyzed data as it were collected. The aim of our analysis was to draw out findings that could inform content and improve design from the perspective of users and stakeholders. Drawing from the user experience framework, we sought in particular to identify issues regarding usefulness, usability (ease of use), and understandability.^17^ Rather than performing formal qualitative analyses, we adhered to a quicker method of identifying problems as they emerged and rapidly trying out solutions in new prototypes.^23^, ^24^

The teams shared a common approach to processing data from piloting and feedback collection, based on the detailed method description in the project protocol^6^ and modifying it as needed. This shared approach consisted of a set of steps: (1) based on notes, interview transcripts, or oral description (e.g., discussions immediately following a workshop), one or more researchers identified problems that users or stakeholders had demonstrated or expressed and grouped them according to framework feature (e.g., “judgment response option – difficulty understanding the meaning of “varies””); (2) one or more researchers rated the issue for perceived seriousness for the user experience (those that obstructed intended use or led to serious misunderstandings), according to a predefined scale; (3) after the team agreed which problems were likely most serious, they discussed the underlying issues to which these data pointed; (4) the team then generated ideas that might resolve these underlying issues, sometimes drawing on direct suggestions from users and stakeholders, while taking care to maintain consistency with the rest of the content or design; (5) one or more ideas were discussed by the team leaders in their meetings and incorporated into new prototypes; (6) the process was repeated to test the new solutions. Additionally, teams drew on findings from systematic reviews of criteria used in different kinds of decision making.^1^

We iterated continuously, making small, frequent incremental adjustments more often than major changes. In Phase 1, teams experimented with diverse approaches to content and format, but recognized early in the process the strengths of establishing a common core. When teams reached consensus regarding EtD content and structure, we created a common prototype to be used by all teams and that formed the basis of the remaining development. In Phase 2, the cycles of feedback and prototyping of the iEtD tool was coordinated and carried out by the iEtD tool team. Additionally, all four teams continued to test and collect feedback on the paper‐based EtD frameworks, passing relevant findings on to the iEtD tool team.

## 3 Results

### 3.1 Phase 1 – EtD Framework Development

#### 3.1.1 Developing a Set of Design Principles and Key Formatting Concerns

Following some early prototyping and testing of example content, we observed actual decision making panels to better understand the context of use (see Additional file S3 in the Supporting Information). Chairs, for instance, faced challenges in managing time, dealing with domineering participants and ensuring information is introduced and discussed in an unbiased way. Panel participants displayed different levels of skills in understanding numerical data and other aspects of research evidence. The large amounts of information appeared to be overwhelming for some, and some panel members were seen to be reading written material rather than following central discussions. Drawing on these observations in addition to findings from earlier work on making evidence understandable and useful in decision making,^17^, ^25^, ^26^ and basic principles for document design,^27^, ^28^ we defined a set of principles to guide design decisions for EtD frameworks.

3.1.1.1

The EtD frameworks need to:

(1) Present the evidence and judgments in a way that is understandable to people without technical expertise;
(2) Have a coherent, logical, visible structure;
(3) Not be longer or more complex than necessary;
(4) Keep relevant information collected close together on the same page;
(5) Present content using a layered approach, with summary/key points in the top layer and more details on demand.

We entered different types of content into prototypes (“example testing”), to test their flexibility and suitability for different kinds of questions. Our experiences and stakeholder feedback to early prototypes led to a better understanding of some key formatting considerations, including the need to:

(1) Separate judgments about evidence from the evidence itself, and make both explicit;
(2) Keep evidence summaries very compact so they are not overwhelming and provide links to more detailed information;
(3) Be able to consider multiple intervention options;
(4) Be flexible in use (e.g., recognizing that it would not always be feasible or useful to find evidence for all of the criteria or to make judgments about all of the criteria);
(5) Be able to adapt output for different end users, such as decision makers at a local level or health professionals.

We carried out prototyping of content and structure for approximately two years (Figure 1).

#### 3.1.2 EtD Framework – A Common Structural Format and Core Content

**Figure 3:**
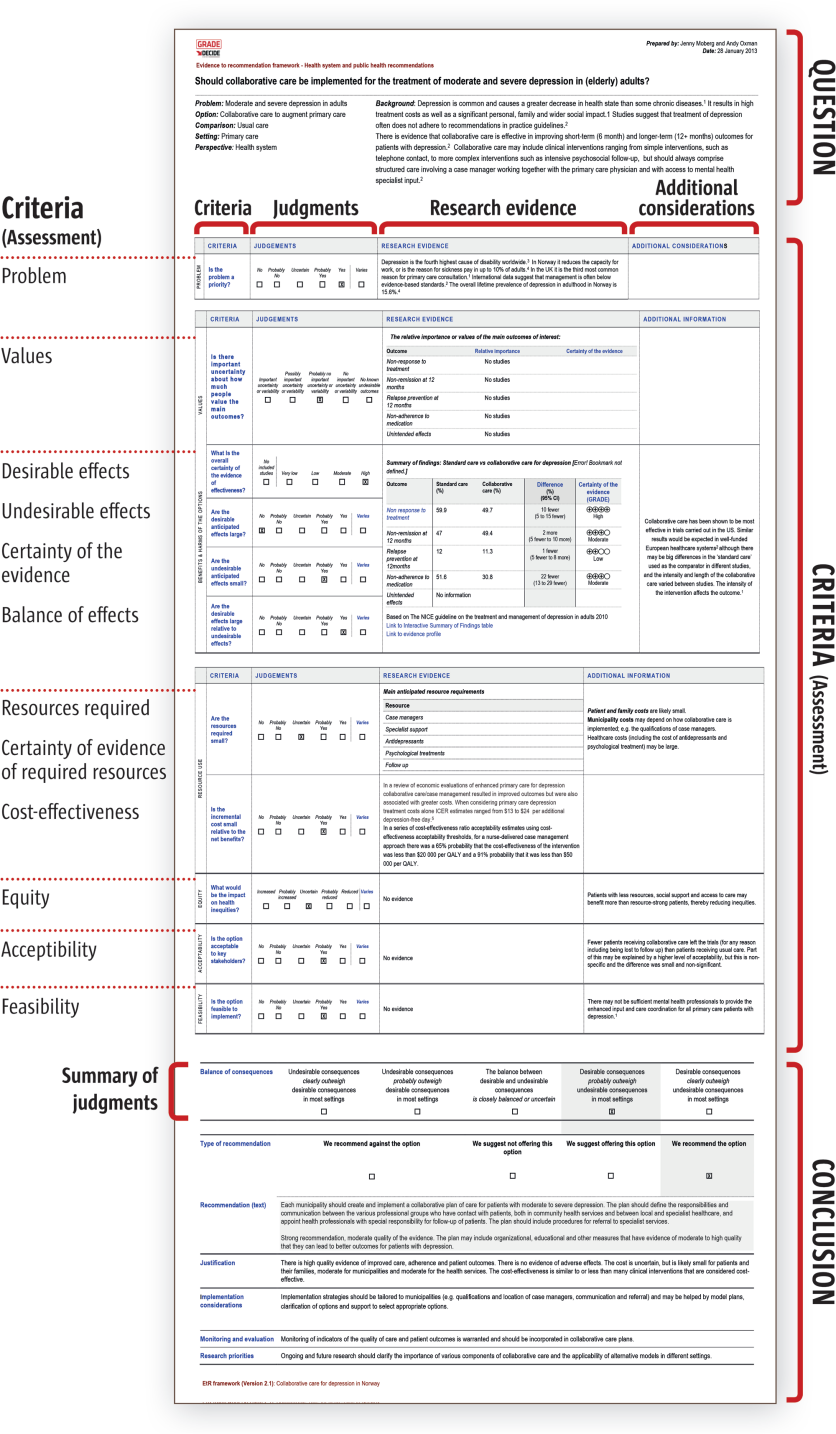
EtD framework paper prototype (mock‐content, not for use).

By the end of Phase 1 teams had reached consensus on a basic EtD structure (**Figure** **3**) and core content for EtD frameworks.^26^ Building on this, teams created topic‐specific EtD versions for decisions concerning clinical interventions, tests, coverage, and health systems and public health options.^29^, ^30^, ^31^, ^32^ These included versions for both recommendations and decisions, from different perspectives (e.g., population or individual perspective) and for consideration of multiple interventions or management options (see Additional file S4 in the Supporting Information). We drew up a set of explanations of terms and concepts used across frameworks that users could access through links in the text.

#### 3.1.3 Main Sections of the EtD

**Question**: The Population, Intervention, Comparison, and Main outcomes (PICO) that the recommendation will address as well as Setting, Perspective, Subgroups, and Background.

**Criteria**: Criteria are the factors that should be considered when making a decision (also see below). For each criterion, the framework offers an opportunity to fill in the following information:

(1) **Judgment**—the option chosen by the panel that reflects their judgment with regards to the specific criterion;
(2) **Research evidence**—evidence that is collected in a preplanned and rigorous fashion to inform a judgment, e.g., evidence from systematic reviews;
(3) **Additional considerations**—other information and considerations to inform or justify each judgment, e.g., practical experience.

**Conclusion**: This includes the summary of judgments, strength of recommendation, recommendation text, justification, implementation considerations, monitoring and evaluation, and research needs.

#### 3.1.4 Criteria

This is the core list from which frameworks for different topics and perspectives are tailored:

- **Problem** – Is the problem a priority?
- **Desirable effects** – How substantial are the desirable anticipated effects?
- **Undesirable effects** – How substantial are the undesirable anticipated effects?
- **Certainty of the evidence of effects** – What is the overall certainty of the evidence of effects?
- **Values** – Is there important uncertainty about or variability in how much people value the main outcomes?
- **Balance of effects** – Does the balance between desirable and undesirable effects favor the option or the comparison (taking the effects, certainty of the evidence, and values into consideration)?
- **Resources required** – How large are the resource requirements (costs)?
- **Certainty of evidence of required resources** – What is the certainty of the evidence of resource requirements (costs)?
- **Cost‐effectiveness** – Does the cost‐effectiveness of the option favor the option or the comparison?
- **Equity** – What would be the impact on health equity?
- **Acceptability** – Is the option acceptable to key stakeholders?
- **Feasibility** – Is the option feasible to implement?

### 3.2 Phase 1 – EtD Framework Feedback

The following are the main thematic findings from Phase 1. These are loosely organized according to three central user experience themes of usefulness, understandability, and usability for different types of users and stakeholders. The findings we have chosen to report here have either been important for development in Phase 1, formed a basis for the work in Phase 2, or are potentially relevant for organizations considering use of the frameworks.

#### 3.2.1 The EtD Is Useful for Structuring Information and Discussion

The most consistent response across the broad range of users and stakeholders who gave feedback was that the framework was perceived as useful and relevant.

*“It is logically organized and puts the key evidence into the context of a decision problem. This is much more relevant and engaging than simply presenting summaries of effect sizes, ICERs, etc.” (Member of stakeholder network)*

Panel chairs valued the EtD framework as a useful tool for managing discussions, teasing out complex issues, and ensuring conclusions were grounded in available evidence. The structure helped them to keep panels on track and limit digressions.

*“It helps … keep to the agenda… (I) have a good excuse to cut people off then, without being impolite” (Guideline developer ‐ chair)*

Chairs reported that use of the EtD helped them limit panel requests to add new evidence to the discussion, and allowed them to “land” judgment discussions or “park” comments as pertaining to criteria other than the one under discussion. Several people emphasized that the key factor for successful use of the framework was a knowledgeable trained chair (or facilitator) familiar with GRADE and with the EtD framework.

*“…if you have a strong chair you can structure the conversation. You can park things where they belong and make the distinction between evidence versus anecdote and make the distinction between effectiveness versus resource use versus acceptability…” (Guideline developer ‐ technical team)*

Panel and workshop participants reported that the separation and organization of judgments and evidence was useful, since it made the relation between these clearer and more explicit. They also appreciated the physical placement of short summaries of evidence on the same page next to the judgment options.

*“I love the formatting… the way evidence to support the answer runs parallel to it.” (User test participant ‐ physician)*

People were positive about an approach that considered both evidence on health effect and evidence on other factors (e.g., acceptability, feasibility).^20^ Several participants also viewed the “Additional considerations” section as useful for including other important sources of information without losing track of or watering down the intention to inform decisions with the best available research evidence.

*“For Resource use, the panel will only have ‘additional information' as opposed to direct evidence. The panel then needs to decide how much weight to put on this information.” (Guideline developer ‐ technical lead)*

Even when no evidence was provided for some of the criteria, participants perceived the framework to be helpful as a checklist to structure meeting discussions.

#### 3.2.2 Understandability Partly Depends on the Skills and Knowledge of Chairs and Technical Teams

**Figure 4:**
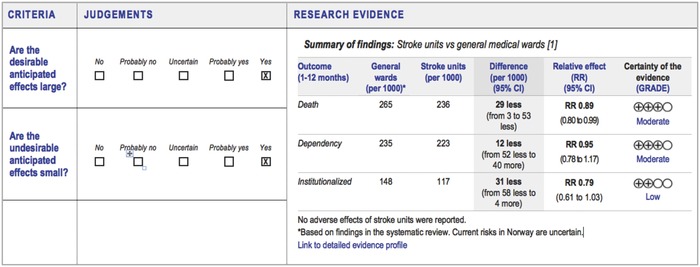
Example of layered approach – the summarized evidence in a condensed format (on right) is the “top layer,” with blue links to next layer of explanations and more detailed information (mock‐content, not for use).

We know from previous work that understandability of evidence is helped by presenting main findings in a concise format, with access to more details elsewhere.^33^ We designed the framework using this layered approach (**Figure** **4**) that can help make the document less overwhelming, more understandable, and can potentially help level the playing field for nonexpert participation in discussions. However, the chairs and technical teams found preparing condensed presentations of evidence challenging and needed additional skills to make this concept work.

*“To try to encourage people who are quiet to speak up… is also tied to the way that you present the evidence ‐ have you presented it in a way that is not intimidating?…A lot of the discussions we had were around sort of crash course in how to present evidence in a very small space.” (Guideline developer ‐ technical team)*

People participating in panels also needed explanations of less familiar elements, such as interpreting the “Values” criterion or judgment options. Linked explanations could resolve some misunderstanding or confusion, but groups also relied heavily on discussions and the knowledge of chair and technical team.

#### 3.2.3 Ease of Use Varied across Respondents

Frameworks were not necessarily easy for technical teams to use. There were many practical implications related to gathering evidence for several criteria. Some were uncertain about where to search for or synthesize “new” types of evidence or what to do when there was no evidence. Guideline producers who used the framework noted that filling in evidence for all the criteria demanded a lot of extra resources and new skills, and emphasized the need for guidance and training.

*“The framework adds another layer of work… in addition to doing the reviews you kind of have to condense the information and put it into this thing in a way that makes sense to other people to use in the guideline process.” (Guideline developer ‐ technical team)*

Some technical teams experienced trouble deciding what criterion an issue belonged to. Others pointed out that placement was less important as long as the issue was covered somewhere.

Though the structure and purpose appeared to be intuitive to panel members, they did not always experience it as easy to use. For instance, choosing among a set of given judgment options (e.g., “trivial” or “large” effect) could be challenging, and led to much careful consideration of options and wording by the development teams.

Feedback indicated that people were divided in their views about how much detail was desirable. Although the prevailing view was that the framework accurately reflected the complexity of decision making, some wanted a simpler solution, while others requested more complexity.

#### 3.2.4 Organizations Have Their Own Processes and Work Flows, and Need Flexibility

Several guideline producers expressed a desire to tailor the frameworks to meet their mandate and perceived needs by adding or deleting criteria. For instance, in an evaluation of the framework, the Swedish Institute of Public Health suggested including Sustainability (which we included as a detailed consideration under “Feasibility”) and Autonomy (which we included as a detailed consideration under “Acceptability”) as separate criteria.^34^ People also wanted be able to change the judgment options, both to fit different types of guideline questions and also to fit guideline producers' standards.

We also observed that guideline producers used EtD frameworks in very different ways. In one project, the technical team filled out entire frameworks for a large set of questions beforehand, including tentative judgments and draft recommendations, saving the panel meeting time to focus on areas of disagreement. In other projects with fewer questions, panels filled in all the judgments during the panel meeting. In yet another organization, the chair used the list of criteria as a checklist for discussion, without filling in each criterion's judgments explicitly.

### 3.3 Phase 2 – iEtD Tool Development

In Phase 2, the iEtD tool team turned their attention to the question of how we might facilitate preparation and use of EtD frameworks with an online tool.

**Figure 5:**
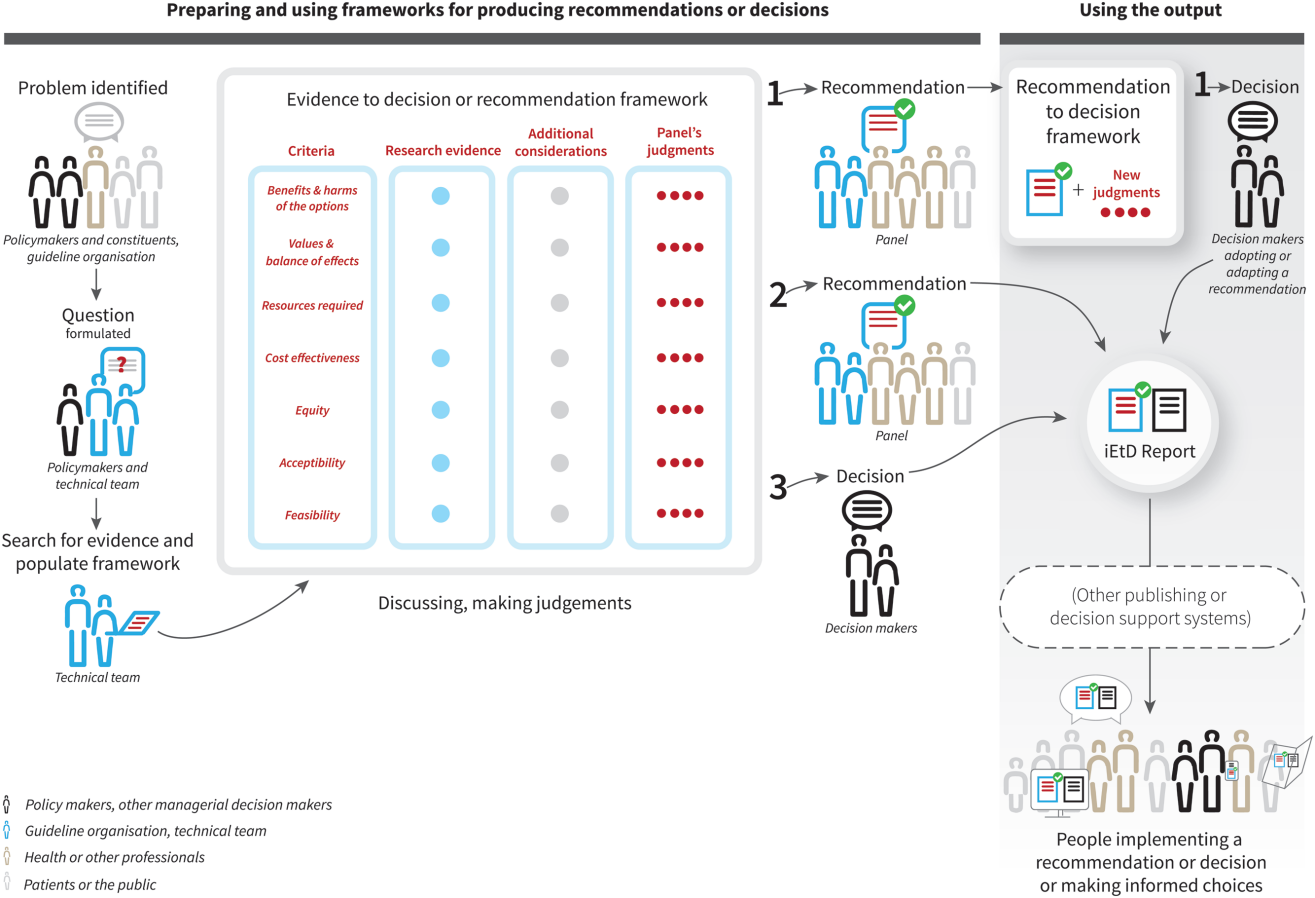
A conceptual map of producing and using iEtD frameworks, for moving from (1) Recommendations to Decisions, (2) Evidence to Recommendations, or (3) Evidence to Decisions.

The tool should support three types of guideline and decision making processes: moving from (1) Recommendations to Decisions, (2) Evidence to Recommendations, or (3) Evidence to Decisions (**Figure** **5**).

#### 3.3.1 Additional Set of Design Principles

We supplemented the design principles established in Phase 1 for EtD development with principles grounded in guidelines for web usability^35^ and based on feedback and other findings from Phase 1.

##### 3.3.1.1 Additional Principles for Guiding Design Decisions for iEtD Tool

(1) Simple intuitive “what you see is what you get” text entry;
(2) Minimal need for training;
(3) Full user control (e.g., no mandatory order of steps or fields that must be completed in order to move on; user control of what content is included when exporting);
(4) Suitable for use in groups (across all phases of use);
(5) Suitable for screen projection, computer viewing, or printing on paper;
(6) Maximal flexibility, for use by different people, for different target audiences, and in different organizations or work flows;
(7) Open access.

Although frameworks can be used many types of groups making various kinds of decisions, supporting guideline development was a central focus in the DECIDE project. Therefore, in defining user roles and tasks in order to specify iEtD functionality, we modeled use of the tool on our knowledge of evidence‐informed guideline processes, drawing on experience from DECIDE partners.

#### 3.3.2 Establishing Desired Functionality

**Figure 6:**
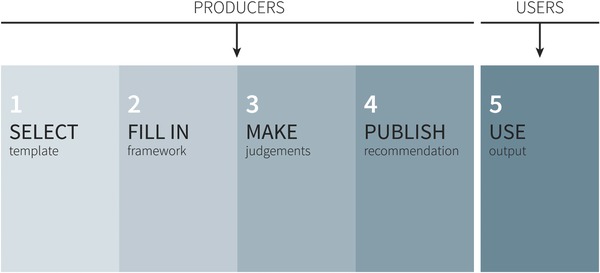
Five main steps for preparing and using frameworks in the iEtD tool.

To maintain framework consistency across multiple users, we determined that people preparing frameworks would choose from a list of standardized templates and enter content in fixed text fields. Creating and using EtD templates in a guideline process therefore involved five basic steps: selecting (or tailoring) a template, filling in a template, making judgments and a recommendation, publishing a recommendation, and using the recommendation (**Figure** **6**).

We also defined a set of roles that represent people who either produce or use guidelines: the organization (e.g., guideline owner), technical team (e.g., research staff and project manager), panel chairperson, panel, and end users (people who read the published recommendation or use it as a basis for a decision).

Starting with these five steps and roles, and drawing on our teams' experience in guideline development processes and on feedback from Phase 1, we generated ideas about what kind of detailed tasks people in these different roles would need to be able to do during the different steps. This led to a list of desired functionality that formed the basis of our design (see Additional file S5 in the Supporting Information).

#### 3.3.3 iEtD Tool – Main Features

**Figure 7:**
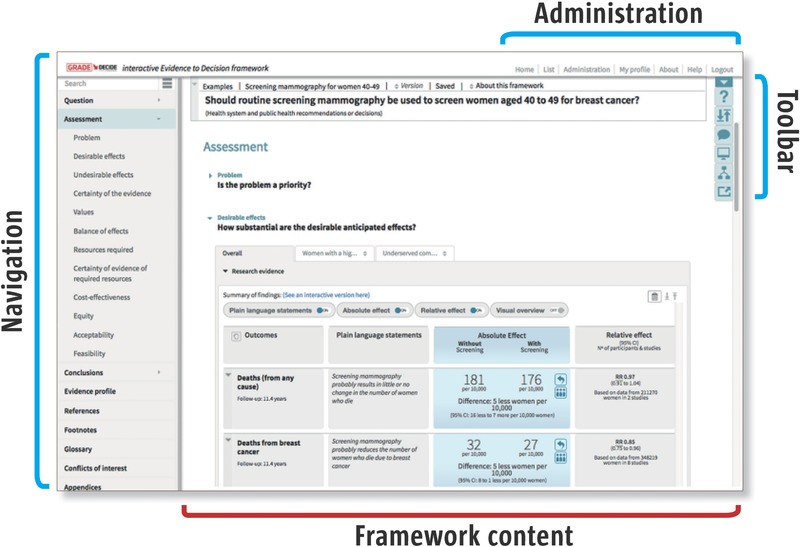
Screenshot of the iEtD tool.

The iEtD tool includes all the content from EtD frameworks, in addition to interactive functionality for tailoring, preparing, administrating, or using the content (**Figure** **7**). It is designed for collaborative, online use.

In addition to the main parts of the EtD (“Question, Criteria, Conclusion”), we added new sections for more detail (e.g., Project management, Evidence profiles, Footnotes, References, Conflicts of interest). We added two new fields under each criterion: Detailed judgments and Panel discussion. Detailed judgments are topic‐specific sets of questions that can help panels unpack the issues underlying a judgment when there is disagreement or when the judgment is difficult to make. We added Panel discussion based on concerns from one organization reporting that some panels felt their role was reduced to mechanically approving prechecked judgment option boxes, and that the framework lacked a space for recording panel discussions they considered important.

We created guidance for producers of EtD frameworks^36^ and developed functional guidance for explaining how to use the tool. We added a short glossary of terms used in the tool.

We established anonymous, optional voting functionality in the judgments and conclusions sections. We created a toolbar with functionality for managing different viewing options (e.g., tailored for projection in a meeting room), finding contextually relevant guidance, making comments, and exporting. We created a top menu with administrative functionality, such as organizing projects, templates, and users and version control (see Figure 7). We made sure that it is possible to use the tool offline.

We developed templates for exporting to print and digital formats (Word, PDF, html). This included both templates for draft reports and for final publications aimed at end users. For the latter, we achieved a layered approach by reordering the framework sections so that the conclusion came first. We also created templates for “Recommendations to Decisions” for people making decisions based on recommendations (e.g., policy makers making a national decision based on an international recommendation).

We implemented several features that enable organizations to tailor the iEtD. Templates for creating or exporting EtD frameworks are editable: criteria can be added or removed, response options (the range of answers provided for making judgments and detailed judgments) can be changed, text in the guidance section can be edited (so that organizations can create tailored instructions), and all template text can be rewritten in another language. Organizations can also add their logo. People who are registered as an organization administrator can accept members to their projects and assign them any functionality (e.g., editing, commenting, voting).^37^

### 3.4 Phase 2 – iEtD Tool Feedback

Much of the feedback described in Phase 1 is applicable to use and preparation of EtD frameworks regardless of whether they are in a paper or digital format. Here, we present feedback and observations that are specifically relevant to preparation and use of frameworks in a digital format created with the iEtD tool.

#### 3.4.1 Barriers to Using an Unfamiliar Digital Tool

Most workshop or training attendees found the iEtD intuitive and easy to learn. However, others felt more comfortable using the paper‐based framework prototypes from Phase 1. Some technical teams felt that learning to use unfamiliar technology placed an unnecessary extra stress on their already overloaded agendas.

*“Everything that is digital is a barrier ‐ learning to use it takes time.” (Guideline program manager)*

One guideline program manager noted that they constantly had newcomers joining their teams who needed to learn many other things at the same time, such as GRADE methodology. This lowered their ability or desire to engage with new technology at the same time.

Some feedback also indicated that people had mistaken expectations that the digital tool would produce automatically calculated conclusions.

#### 3.4.2 Concerns about Using an External System

Some people had concerns related to IT security and ownership. People who were unfamiliar with the system host (Epistemonikos) were uncertain whether they could trust that their data were backed up and secure. Some said their organization would not permit them to use a system that was hosted on an external server. There was concern about the need for a stable internet connection or making sure nothing technical would go wrong when running time‐pressed panel meetings.

#### 3.4.3 Interactive Voting Appeared to Be a Useful Discussion Tool

Interactive, anonymous voting appeared to provide a useful method of charting the groups' distribution of differing judgments without identifying the views of specific individuals. In workshops, we observed groups using voting to spark discussion rather than to just finalize conclusions, providing a starting point for presenting different opinions and clarifying misinterpretations of the evidence.

## 4 Discussion

Using a human‐centered design approach, we created a common core structure and terminology for EtD frameworks and developed topic‐specific variations of frameworks for decisions about clinical interventions (treatments) and tests, coverage, and health system or public health interventions. We developed a flexible iEtD tool for producing, using, and adapting transparent frameworks in decision making and communication. Stakeholders and users experienced the EtD frameworks as a useful reminder of the most important criteria for making decisions and for bringing evidence into the decision making in a systematic way that helped bring clarity and structure to their thinking, discussion, and dissemination. The layered format, which entails use of highly condensed evidence, was key to understanding of evidence and to ease of use in discussions, but requires skill to prepare. Populating frameworks, learning to use the digital tool, and helping panels demanded extra resources and skills that organizations may lack. The iEtD tool has several unique features, such as interactive voting that help groups identify reasons for disagreement, and export formats that are tailored for different end users and that can facilitate understanding, implementation, revision, or adaption.

### 4.1 Strengths and Limitations

The strength of this work was the large amount of feedback provided throughout the project, by a wide range of stakeholders and users from multiple perspectives and for several types of decision making. Frameworks underwent rigorous user‐testing and iterative development, as well as real‐life testing with guideline panels. The multidisciplinary team brought a rich set of skills and perspectives to the development.

One limitation was fewer cycles of prototyping and feedback in Phase 2 due to the time needed for software development. Lack of time at the end of the project also limited the number of export format iterations and amount of end user feedback about these that we could carry out. However, we drew heavily on earlier work exploring effective ways to support both health professionals' and consumers' understanding of evidence,^25^, ^38^ as well as concurrent work in the DECIDE project exploring both of these groups' user experiences of output from clinical practice guidelines.^39^, ^40^

### 4.2 EtD and iEtD: Examples of Technology Designed to Link Knowledge to Action

*“Efforts to mobilize S&T (science and technology) for sustainability are more likely to be effective when they manage boundaries between knowledge and action in ways that simultaneously enhance the salience, credibility, and legitimacy of the information they produce.”* 41

The EtD framework and iEtD tool are examples of technologies built to help link knowledge to action. Technologies are likely to be effective when the information they produce is salient, credible, and legitimate.^42^, ^43^, ^44^ A significant challenge is that these attributes can mean different things to different stakeholders. The iEtD is designed to enable more voices to actively participate in decision making processes, by rendering the criteria, the evidence, and rationales for judgments easier to understand. This can increase the legitimacy of the process for end users of the decision. Additionally, output is tailored for different groups of end users using it for different purposes. Importantly, end users are not conceptualized as passive recipients of top‐down dissemination efforts, but as potentially active participants who may need or want to reconsider the individual judgments, conclusions, and meaning of the output for their own context.

### 4.3 Designing to Improve Communication

In implementation science, including guideline dissemination, communication is commonly conceptualized as “information transfer” – packaging information (such as recommendations and evidence) in ways that make it clear and understandable for a target audience.^45^ However, in complex systems with many stakeholders, perspectives, and concerns, communication is more complicated and can break down rendering an “information transfer” approach insufficient. Instead, communication needs to be viewed as a process of creating “shared understanding” within and between groups.^45^

In this project, we worked along both of these dimensions. Some of our design efforts concentrated on enabling successful “information transfer” by improving the individual user's experience of the information (e.g., increased understandability, usability, and usefulness of the information). But, we also worked to enhance group communication and “shared understanding” within SACs and between many groups, by working to create transparent solutions that could support discussion, participation, and mediation. The EtD and iEtD can support improved communication:

(1) Within technical teams while they are creating and editing framework content;
(2) Within SAC panels while they are discussing framework content;
(3) Between organizations, technical teams, and SAC panels;
(4) Between organizations and end users of framework output.

### 4.4 For What Contexts May EtD and iEtD Be Most Useful?

Many organizations still struggle with overdependence on “expert opinion,” often resulting in recommendations or decisions that are unbalanced and not possible to retroactively unpack and inspect. Although the EtD and iEtD have been designed specifically for decisions about health care intervention options, they provide an example for any sector of how evidence can be brought to decision making in a transparent and systematic manner and how experts might assume a more balanced role in SACs together with other stakeholders. EtD and iEtD can also be tailored for use in other sectors to answer intervention questions that have a PICO structure (population, intervention, comparison, outcome).

At first glance, use of EtD and iEtD may appear suitable only for use in projects with ample time and resources for producing comprehensive systematic reviews of evidence for each criterion. However, they can also be used in contexts even where there is little or no evidence. Using the framework, the lack of evidence can be made apparent, allowing readers to see which other considerations informed the judgments and conclusions. The iEtD may be more useful in organizations where there is a stable pool of technical team staff, who can develop the skills needed to use the technology.

EtD and iEtD also have the potential to be used as teaching tools, for instance, to train medical students or other groups in systematic approaches to collaborative decision making or prepare them for participation in civil society. We are planning to use iEtD in future work as part of an ongoing effort to teach young people how to make informed health choices.^46^

### 4.5 Future Development and Research

Exploring how best to link knowledge to action across multiple stakeholders requires a research approach that looks at a wider set of issues than individual users' interactions with a piece of information or technology interface. Future development of the EtD and iEtD should be accompanied by research questions and methods that more specifically explore how the technology can best support communication between producers, panels, and users. Work should also be carried out to explore how use affects the participation of panel members, their conclusions, the transparency of reports for end users, and the usefulness of that output for those audiences. The potential for adaptation of this approach to multistakeholder decision making for other sectors as well as across sectors – an important consideration in relation to the recently agreed Sustainable Development Goals^47^ – could also be considered.

### 4.6 Access and Additional Tools

According to recent estimates, there are currently over 7000 users of the static and interactive versions of the EtD frameworks. More detail about each type of EtD framework and links to other relevant tools can be found in Additional file S4 (Supporting Information).

The stand‐alone, open access iEtD tool^48^ was codeveloped and programmed by Epistemonikos,^49^ who continues to host and maintain the system. It is available for free noncommercial use: https://ietd.epistemonikos.org.

EtD frameworks are also accessible through GRADEpro Guideline Development Tool^50^ that hosts an iEtD tool in a one stop solution for guidelines and decisions. It is free for noncommercial use: https://gradepro.org (www.gradepro.org).

## Conflict of Interest

The authors declare no conflict of interest.

## Supporting information

Supplementary
