# Supplementary material for: Developing Evidence to Decision Frameworks and an Interactive Evidence to Decision Tool for Making and Using Decisions and Recommendations in Health Care
Source: Glob Chall. 2018 Jan 10;2(9):1700081. doi: 10.1002/gch2.201700081 (PMC6607226; doi:10.1002/gch2.201700081)
Supplement: Supplementary file 1 — Supplementary [file GCH2-2-1700081-s001.pdf]

Copyright WILEY-VCH Verlag GmbH & Co. KGaA, 69469 Weinheim, Germany, 2017.

Global Challenges
Open Access

Supporting Information

for *Global Challenges,* DOI: 10.1002/gch2.201700081

Developing Evidence to Decision Frameworks and an Interactive Evidence to Decision Tool for Making and Using Decisions and Recommendations in Health Care

Sarah E. Rosenbaum, Jenny Moberg, Claire Glenton, Holger J. Schünemann, Simon Lewin, Elie Akl, Reem A. Mustafa, Angela Morelli, Joshua P. Vogel, Pablo Alonso-Coello, Gabriel Rada, Juan Vásquez, Elena Parmelli, A. Metin Gülmezoglu, Signe A. Flottorp, and Andrew D. Oxman*

Supporting Information

Developing Evidence to Decision Frameworks and an Interactive Evidence to Decision Tool for Making and Using Decisions and Recommendations in Health Care

Sarah E. Rosenbaum, Jenny Moberg, Claire Glenton, Holger J. Schünemann, Simon Lewin, Elie Akl, Reem A. Mustafa, Angela Morelli, Joshua P. Vogel, Pablo Alonso-Coello, Gabriel Rada, Juan Vásquez, Elena Parmelli, A. Metin Gülmezoglu, Signe A. Flottorp, and Andrew D. Oxman*

Additional file 1: *Details about range of methods used in cycles of development*

Additional file 2: *Additional file 2: List of user-test interviews and structured feedback sessions from 2012-2015*

Additional file 3: *Description of a panel discussion*

Additional file 4: *Overview of the EtD frameworks and links to tools*

Additional file 5: *User roles and tasks*

Additional file 1: *Details about range of methods used in cycles of development*

**Aim**	**Method**	**Types of users and stakeholders** *(and project team members)*	**Format/ Location**	**More detail***
**PROTOTYPING**				
Reviewing systematic reviews of decision making frameworks, sets of criteria	*Reading and discussing*	*(Project teams)*	Project office and meetings	BMJ, 2016 [1]
Experiment with what elements to include and how to format and structure them, what labels and wording to use	*Paper prototype sketching*	*(Project teams)*	Project office and meetings	
Experiment how well prototypes worked for different types of examples and content	*Example testing*	*(Project teams)*	Project office and meetings	
Exploring visual, interactive and system solutions (Phase 2)	*Sketches, prototyping, software development*	*(iEtD tool team)*	Programs for designing and coding	
**PILOTING AND FEEDBACK COLLECTION**				
Determine policymakers' perceptions regarding the criteria in the EtD framework and how best to summarise and present evidence to support health system decisions	*Survey*	Health system policy makers and managers from 15 countries and the World Health Organization	Online question-naire	Health Res Policy Syst, 2013 [2, 3]
Assess the applicability of the EtD framework for coverage decisions to the United States	*Survey and workshop feedback*	Representatives of US-based organizations making coverage decisions from the Centers for Medicare and Medicaid Services (CMS) and private insurers, stakeholders from Canada, Great Britain, and Australia	Workshop	J Clin Epidemiol. 2017 [3]
Gain insight into a typical use context	*Non-participatory observation*	International guideline panels who were not using EtD frameworks, WHO *(& Observers from project teams)*	Guideline meetings	(See *Additional file 3*)
Gain insight into problem areas, identify barriers and facilitators, see opportunities for improvement	*Piloting in guideline projects, participatory observation*	International and national guideline panels who used EtD framework in actual guideline production in WHO, WAO, Saudi Arabia, Colombia, Spain *(& Chairs, technical teams and observers from project teams)*	Guideline meetings:	Implement Sci. 2015 [4]<br><br>Implement Sci. 2015

				[5]
	Participatory observation of mock panels	Health professionals, decision/policy makers/managers, guideline producers, and researchers at international conferences or workshops learning about EtD frameworks *(& Chairs and observers from project teams)*	Training or conference workshops	
Collect feedback about user and stakeholder needs, concerns, experiences, barriers and facilitators, suggestions for improvement	*User-test interviews*	International guideline panel members and chairperson, technical teams, WHO and Europe; Health professionals, policymakers, guideline chair, researchers from > 10 countries; EBM workshop participants including journalists and policy makers from Europe and USA *(Interviewers from project teams)*	Face-to-face or online sessions, during guideline meeting recesses and workshops	*(See Additional File 2)* Implementation Sci. 2013 [6]
	Stakeholder feedback	International advisory group network of policy makers, health professionals, guideline producers, and researchers, as well as GRADE working group members not directly participating on project teams	Email, face-to-face meetings	
	Structured feedback	International and national guideline panels who used EtD framework in actual guideline production in WHO, WAO, Saudi Arabia; policy makers, health professionals, guideline producers, and researchers participating in workshops in multiple countries and international conferences	Question-naires	*(See Additional File 2)* Implement Sci. 2015 [5] J Clin Epidemiol. 2017 [7]
Discuss perceived organizational or contextual barriers and facilitators when implementing or adapting EtD, iEtD or output from iEtD	*Discussions*	Guideline producer (WHO) and health policymakers from seven Eastern European countries (not partners in DECIDE) *(Facilitators and observers from project teams)*	Face-to-face meetings	*(See Additional File 2)*
Assess applicability of EtD framework applicable for public health decisions in Sweden.	*External evaluation***	Researchers in Swedish national institute of health (not partners in DECIDE)	Evaluation at Swedish national institute of health	Health Promot Int. 2016 [8]

*Some methods and/or results are written up in more detail in individual articles
** Single evaluation, initiated and carried out independently outside of the project

Additional file 2: *List of user-test interviews and structured feedback sessions from 2012-2015*

Tested what	How many participants?	What kind of session?	Type of participants	Where
**Work Package 1 - Health professionals**				
EtD output for clinical decisions, Top layer	7	Individual with ipad/iphone	Physicians	Norway, Spain, Canada, Scotland
	3 groups	Group session with ppt/images	Physicians	Norway, Spain, Canada
	1 group	Group session	Physicians	Finland
	18	Individual and Survey	Physicians	Norway, Spain, UK, USA, Canada, Saudi Arabia
**Work Package 2 – Coverage**				
EtD framework	1 group	Group session with feedback questionnaire	Regional Commission for Drugs Coverage	Italy
	1 group	Presentation with feedback	Stakeholder organization representatives	USA
	2 groups	Feedback questionnaires	Participants at Cochrane & HTA conferences	Spain, Italy
	8	Individual	Managers and policy maker (with medical and economics background)	Italy
**Work Package 3 – Patients and public**				
Prototype of guideline output	1 group (5 people)	Groups – divided into pairs with ppt booklet	Young people	Scotland
	5	Individual interviews	Homeless with low literacy	Scotland
	2 groups (9 & 8 people)	Group – divided into pairs with ppt booklet	University staff and members of public	Scotland
	2 groups (approx. 10 & 10 people)	Group session, pairs with ppt booklet	Mostly physicians (GP), also dentists, members of public	Scotland
**Work Package 4 – Diagnostic tests and screening**				
EtD framework	6 small groups	Group sessions with a note taker documenting the feedback	GL developers, systematic reviewers, Clinicians	Germany (GIN meeting)
	3	Individuals	GL developers	Different GRADE workshops
**Work Package 5 – Health systems and public health**				
EtD framework	6	Individual	Guideline panelists	Switzerland (WHO)
	1 group	Pilot use with observation and feedback	Guideline panel	Switzerland (WHO)
	1 group	Pilot use with observation	Guideline panel	Switzerland (WHO)

		and questionnaire		
	1 group	Pilot use with questionnaire	Stakeholders in health prof. education (Health prof education)	USA
	1 group (22)	Group session – with survey	Policy makers + technical team in public health	Sweden (workshop in Malta
	5	Individual	Policy makers and journalists	USA (EBHC workshop)
	1 group (30?)	Group session with feedback questionnaire	Clinicians, Policy makers, Journalists at EBHC workshop	USA
Symbols	10	Individual with participant sketching	Researchers and Communications specialists	Norway
iEtD tool	1	Individual	National Medicine Agency	Norway
	1	Individual	Guideline chair/facilitator (European)	Edinburgh (international conference)
	3	Individual	Guideline tech team (Norway and WHO)	Norway
	10 small groups	Group	Mixed	Spain
	1 group (30?)	Group session with feedback questionnaire	Mixed	Edinburgh (international conference)
	Several groups	3-day workshop creating rapid and full HTA	HTA developers	Colombia
EtD & iEtD output for policy makers, (moving from a recommendati on to national decision, using WHO guideline as case)	1-3 representatives from 7 countries & WHO	Groups, 3-day workshop	Health policy makers	Slovenia, Ukraine, Bulgaria, Montenegro, Moldova, Romania, Kosovo

Additional file 3: *Description of a panel discussion*

The following description was reconstructed based on notes from non-participatory observations of meetings with panels not using an EtD framework.

About 20 people from different countries, sitting at long tables in a U-shape. Everyone has a thick binder filled with documentation for the meeting. Some are reading in the beginning of the binder, some are reading in the middle, others are looking or typing at their computer. About half of the people are looking up, listening to the meeting chair who is presenting data projected onto a screen. There is a lot of light coming in from the window at the side of the room, creating poor color contrast and diminishing text legibility, particularly at the back of the room where the distance to the screen makes the text size very small.

One woman raises her hand to ask a question about what is on the screen. She has already spoken up several times. Some people have not spoken yet, and some do not speak publicly during the whole meeting (which lasts two days). The same person holds a document, and says that the others should look at this new report, which has data that conflicts with what is being presented. Some people look thoughtful. Others look in their binder.

The group has several dozen different questions to discuss in their two-day meeting. They spend about 3 hours of the first day on two of the questions, with a lot of time spent on principled discussions about some details that seem disproportionate to the time spent. As the meeting time remaining gets shorter, the group has less and less time for the remaining questions.

In an interview between the first and second day, we ask one of the people who has not spoken up why he is so quiet. He says he does not understand the numerical data so he does not feel confident in speaking up. English is not his first language. He is a doctor with a high-level policy position in his country.

Some of the problems related to use of information illustrated by this description:

- Competing focus of attention (binder/projector screen) making it challenging to collect group focus
- Legibility of projected text on screen
- Huge amount of pre-prepared information
- Some participants are prepared; others are not
- Attempt to introduce new information by one participant
- Very constricted time frame, time management is crucial
- Different levels of English literacy and numeracy among participants, hindering full understanding of information
- Lack of confidence in understanding hindering participation
- Potential for discussion being dominated by individual, outspoken participants

(See Additional File 2)
Overview of the EtD frameworks and links to tools

**Subject**	**Type of framework**	**Reference**
Overview		BMJ, 2016 [1]
Development of the EtD and iEtD		(this article)
**Clinical practice**	Clinical recommendation - Individual perspective<br>Clinical recommendation - Population perspective<br>Clinical recommendation - Individual perspective - Multiple options<br>Clinical recommendations - Population perspective - Multiple options	BMJ, 2016 [9]
**Coverage**	Coverage decision<br>Coverage decision - Multiple options	International Journal of Technology Assessment in Health Care (accepted 2017) [10]
**Tests (e.g. diagnostic, screening)**	Tests - Clinical recommendation - Individual perspective<br>Tests - Clinical recommendation - Individual perspective - Multiple options<br>Tests - Clinical recommendation - Population perspective<br>Tests - Clinical recommendation - Population perspective - Multiple options<br>Tests - Coverage decision<br>Tests - Coverage decision - Multiple options	Journal of Clinical Epidemiology, 2016 [11]
**Health system and public health**	Health system and public health recommendation<br>Health system and public health decision<br>Health system and public health recommendation - Multiple options<br>Health system and public health decision - Multiple options	WHO Bulletin, 2017 (submitted) [12]
Guidance		iEtD.epistemonikos.org [13]

Links to tools

The stand-alone, open access iEtD tool was co-developed and programmed by Epistemonikos, who continues to host and maintain the system. It is available for free non-commercial use: https://ietd.epistemonikos.org

EtD frameworks are also accessible through GRADEpro Guideline Development Tool that hosts an iEtD tool in a one stop solution for guidelines and decisions. It is free for non-commercial use: https://gradepro.org (www.gradepro.org)

We also identified the need for two additional tools during this work, and developed these in parallel:

- interactive Summary of Findings (iSoF) – tool for creating interactive presentations of evidence of effects from systematic reviews using multiple representations (text, numbers, images). iSoF tables can be imported into iEtD frameworks (they are also available through GRADEpro GDT, including an iSoF for medical tests).

- GET IT glossary – free online glossary providing explanations of terminology used in the frameworks in plain language[14]

Additional file 5: *User roles and tasks*

User roles:

- Organisation (e.g. guideline owner)
- Technical team (e.g. research staff and project manager)
- Panel chairperson
- Panel
- End users (people who read the published recommendation or use it as a basis for a decision).

Tasks:

Organizations, technical teams, chairs, panels need to be able to:

- Register, log in
- View frameworks, search within a framework

Organizations, technical teams, chairs need to be able to:

- Administrate Organizations and Users registered to their organization
- Create, edit and manage Projects
- Edit and administrate framework and export Templates
- Create, organize, edit, and track changes in individual Frameworks
- Importing interactive Summary of Findings tables
- Add footnotes, endnotes, references, and a glossary
- Add appendices and information regarding conflicts of interest of panelists
- Present using a projector
- Manage viewing options (who can see what content)

Panels need to:

- Comment on entered content (before and during a panel meeting)
- Discuss, make judgments and vote
- Access guidance, glossary and functional help
- Export reports

End users need to:

- View exported reports
- Make a decision based on a published recommendation (Recommendation to Decision framework), describe what drove that decision if it is different than the recommendation

The tasks described above formed the basis for our interface design and the functionality that the software developers built.

To begin with, we considered re-creating these five user roles in the software development and assign sets of permissions to them, corresponding to this list, in order to allow organizations to have different levels of access for different kinds of users.

However, we moved away from that solution because we observed that organizations work in different ways and needed flexibility with regard to deciding who may do what, for instance edit, comment, vote in a project. Instead we created an administrative page where project administrators could assign desired functionality to any registered user in their project.

1. Alonso-Coello P, Schunemann HJ, Moberg J, Brignardello-Petersen R, Akl EA, Davoli M, Treweek S, Mustafa RA, Rada G, Rosenbaum S, Morelli A, Guyatt GH, Oxman AD, Group GW. GRADE Evidence to Decision (EtD) frameworks: a systematic and transparent approach to making well informed healthcare choices. 1: Introduction. BMJ. 2016 Jun 28;353:i2016.
2. Vogel JP, Oxman AD, Glenton C, Rosenbaum S, Lewin S, Gülmezoglu AM, Souza Jã P. Policymakers' and other stakeholders' perceptions of key considerations for health system decisions and the presentation of evidence to inform those considerations: an international survey. Health Res Policy Syst. 2013;11:19.
3. Dahm P, Oxman AD, Djulbegovic B, Guyatt GH, Murad MH, Amato L, Parmelli E, Davoli M, Morgan RL, Mustafa RA, Sultan S, Falck-Ytter Y, Akl EA, Schunemann HJ. Stakeholders apply the GRADE evidence-to-decision framework to facilitate coverage decisions. J Clin Epidemiol. 2017 Jun;86:129-39.
4. Glenton C, Lewin S, Gülmezoglu AM. Expanding the evidence base for global recommendations on health systems: strengths and challenges of the OptimizeMNH guidance process. Implement Sci. 2015;11.
5. Neumann I, Brignardello-Petersen R, Wiercioch W, Carrasco-Labra A, Cuello C, Akl E, Mustafa RA, Al-Hazzani W, Etxeandia-Ikobaltzeta I, Rojas MX, Falavigna M, Santesso N, Brozek J, Iorio A, Alonso-Coello P, Schunemann HJ. The GRADE evidence-to-decision framework: a report of its testing and application in 15 international guideline panels. Implement Sci. 2016 Jul 15;11:93.
6. Treweek S, Oxman AD, Alderson P, Bossuyt PM, Brandt L, Brozek J, Davoli M, Flottorp S, Harbour R, Hill S, Liberati A, Liira H, Schunemann HJ, Rosenbaum S, Thornton J, Vandvik PO, Alonso-Coello P. Developing and Evaluating Communication Strategies to Support Informed Decisions and Practice Based on Evidence (DECIDE): protocol and preliminary results. Implement Sci. 2013 Jan 09;8:6.
7. Dahm P, Oxman AD, Djulbegovic B, Guyatt GH, Murad MH, Amato L, Parmelli E, Davoli M, Morgan RL, Mustafa RA, Sultan S, Falck-Ytter Y, Akl EA, Schünemann HJ. Applying GRADE to coverage decisions: results of a stakeholder survey and workshop. Journal of Clinical Epidemiology. 2017;pii:(S0895-4356(17)):30335-9.
8. Guldbrandsson K, Stenstrom N, Winzer R. The DECIDE evidence to recommendation framework adapted to the public health field in Sweden. Health Promot Int. 2016 Dec;31(4):749-54.
9. Alonso-Coello P, Oxman AD, Moberg J, Brignardello-Petersen R, Akl EA, Davoli M, Treweek S, Mustafa RA, Vandvik PO, Meerpohl J, Guyatt GH, Schunemann HJ, Group GW. GRADE Evidence to Decision (EtD) frameworks: a systematic and transparent approach to making well informed healthcare choices. 2: Clinical practice guidelines. BMJ. 2016 Jun 30;353:i2089.
10. Parmelli E, Amato L, Oxman A, Alonso-Coello P, Brunetti M, Moberg J, Nonino F, Pregno S, Saitto C, Schünemann H, Davoli M, GRADE Working Group. GRADE Evidence to Decision (EtD) framework for coverage decisions. International Journal of Technology Assessment in Health Care (accepted).
11. Schunemann HJ, Mustafa R, Brozek J, Santesso N, Alonso-Coello P, Guyatt G, Scholten R, Langendam M, Leeflang MM, Akl EA, Singh JA, Meerpohl J, Hultcrantz M, Bossuyt P, Oxman AD, Group GW. GRADE Guidelines: 16. GRADE evidence to decision frameworks for tests in clinical practice and public health. J Clin Epidemiol. 2016 Aug;76:89-98.
12. Moberg J, Oxman AD, Rosenbaum SE, Schunemann HJ, Guyatt G, Flottorp S, Glenton C, Morelli A, Rada G, Alonso-Coello P, for the GRADE Working Group. GRADE

Evidence to Decision (EtD) frameworks for health system and public health decisions. Sumbitted to WHO Bulletin. 2017.
13. Moberg J, Alonso-Coello P, Oxman A. GRADE Evidence to Decision (EtD) Frameworks Guidance. Version 1.1 [updated May 2015]2015.
14. Moberg J, Austvoll-Dahlgren A, Treweek S, Badenoch D, Harbour R, Rosenbaum S, Oxman A, Atkinson P, Chalmers I. A plain language Glossary of Evaluation Terms for Informed Treatment choices (GET-IT) at http://www.getitglossary.org: Norwegian Institute of Public Health2017. Report No.: ISBN 978-82-8082-834-7.
